# Continuity of psychopathology *v.* resilience across the transition to adolescence: role of hair cortisol and sensitive caregiving

**DOI:** 10.1017/S0033291722001350

**Published:** 2023-07

**Authors:** Karen Yirmiya, Shai Motsan, Orna Zagoory-Sharon, Anat Schonblum, Lee Koren, Ruth Feldman

**Affiliations:** 1 Baruch Ivcher School of Psychology, Reichman University, Herzliya, Israel; 2 Faculty of Life Sciences, Bar-Ilan University, Ramat Gan, Israel

**Keywords:** Adolescence, early life stress, hair cortisol concentrations, mother sensitivity, psychopathology, trauma

## Abstract

**Background:**

The transition to adolescence implicates heightened vulnerability alongside increased opportunities for resilience. Contexts of early life stress (ELS) exacerbate risk; still, little research addressed biobehavioral mediators of risk and resilience across the adolescent transition following ELS. Utilizing a unique cohort, we tested biosocial moderators of chronicity in adolescents’ internalizing disorders *v.* resilience.

**Method:**

Families exposed to chronic war-related trauma, *v.* controls, were followed. We utilized data from three time-points framing the adolescent transition: late childhood (*N* = 177, *M*_age_ = 9.3 years ± 1.41), early adolescence (*N* = 111, *M*_age_ = 11 0.66 years ± 1.23), and late adolescence (*N* = 138, *M*_age_ = 15.65 years ± 1.31). In late childhood and late adolescence children's internalizing disorders were diagnosed. At early adolescence maternal and child's hair cortisol concentrations (HCC), maternal sensitivity, and mothers’ post-traumatic symptoms evaluated.

**Results:**

War-exposed children exhibited more internalizing disorders of chronic trajectory and mothers were less sensitive and more symptomatic. Three pathways elucidated the continuity of psychopathology: (a) maternal sensitivity moderated the risk of chronic psychopathology, (b) maternal post-traumatic symptoms mediated continuity of risk, (c) trauma exposure moderated the association between child internalizing disorders at late childhood and maternal HCC, which linked with child HCC. Child HCC linked with maternal post-traumatic symptoms, which were associated with child disorders in late adolescence.

**Conclusion:**

Results demonstrate the complex interplay of maternal and child's biosocial factors as mediators and moderators of risk chronicity across the adolescent transition following trauma. Findings are first to utilize maternal and child's HCC as biomarkers of chronic stress *v.* resilience during adolescence, a period of neural reorganization and personal growth that shapes the individual's lifetime adaptation.

## Introduction

Early life stress (ELS) carries long-term negative consequences for children's well-being, impairing social relationships, stress management, affective processing, and physical and mental health (Cicchetti, 2016; McLaughlin, 2016; O'Connor, Thayer, & Vedhara, 2021). ELS increases the risk for all common psychiatric disorders (Green et al., 2010; Kessler et al., 2010), which often exhibit a persistent pattern throughout life (McLaughlin et al., 2010). Given the heterogeneity of ELS and its deleterious effects, it is important to disentangle effects that are shared by all harsh rearing conditions from those characterizing specific adversities. Furthermore, since most ELS studies use adults’ retrospective account of adverse childhood experiences, it has been recommended that studies utilize physiological measures of chronic stress and pinpoint biosocial mechanisms that may mediate the effects of ELS on development (Lopez et al., 2021). While retrospective studies can shed light on the trajectories leading from ELS to negative outcomes in later life, such accounts are less suitable for elucidating physiological mechanisms that underpin the long-term effects of ELS. For this goal, there is a need to assess links between stress exposure and physiological markers measured at the same time-point or in close temporal proximity.

Among the key pathways by which ELS exerts its long-term influences is the hypothalamic–pituitary–adrenal (HPA) axis and its main biomarker in humans, cortisol (Bunea, Szentágotai-Tătar, & Miu, 2017; van Bodegom, Homberg, & Henckens, 2017; Young et al., 2021). Hair cortisol concentrations (HCC) offers a non-invasive retrospective acount of cortisol secretion over time and correlates with mean salivary cortisol levels measured over long periods (Stalder & Kirschbaum, 2012). The associations between HCC and ELS or trauma exposure during childhood, which marks a specific form of ELS, has been extensively studied over the past two decades; however, results have been mixed (Khoury, Enlow, Plamondon, & Lyons-Ruth, 2019; Russell, Koren, Rieder, & Van Uum, 2012; Stalder et al., 2016; Staufenbiel, Penninx, Spijker, Elzinga, & van Rossum, 2013). While most studies on the associations between HCC with trauma exposure reported increase in HCC among trauma-exposed individuals (Khoury et al., 2019; Stalder et al., 2016), other studies described HPA-axis hypo-activity in the aftermath of trauma, highlighting the need to consider potential moderators in the interpretation of the findings, such as type of stressor, timing effects, or individual and contextual determinants (Khoury et al., 2019; Steudte-Schmiedgen, Kirschbaum, Alexander, & Stalder, 2016).

Most studies on HCC in children and adolescents focused on socioeconomic adversities, maltreatment, or other forms of interpersonal violence, and only few addressed other traumas (Bates, Salsberry, & Ford, 2017; Bryson, Mensah, Goldfeld, Price, & Giallo, 2021a; Bryson, Price, Goldfeld, & Mensah, 2021b; Gray et al., 2018). Similar to adults, correlations were found between HCC and the number of traumatic events in a community-based children sample (Simmons et al., 2016). However, others reported lower HCC in maltreated children (White et al., 2017) or no associations between HCC and measures of stress (Boeckel, Viola, Daruy-Filho, Martinez, & Grassi-Oliveira, 2017; Gerber et al., 2017; McConnell, 2014; Michels, Van De Wiele, & De Henauw, 2017; Villanueva, Montoya-Castilla, & Prado-Gascó, 2017). The relationship between stress and HCC in children may be specific to some adversities but not others. For example, a recent meta-analysis of 44 studies found no association between social adversity and HCC in young children (Bryson et al., 2021a, 2021b), whereas a study on war-exposed Palestinian adolescents reported that HCC was significantly elevated among children suffering from PTSD compared to non-exposed adolescents (Shaheen et al., 2020). These findings underscore the need to disentangle different types of adversity and specify contextual determinants.

Children's stress physiology is highly sensitive to the mother's condition, including her stress response, parenting behavior, and mental state (Martins, Blumenberg, Tovo-Rodrigues, Gonzalez, & Murray, 2020), and it has been shown that maternal warmth and sensitive support can buffer the negative effects of trauma exposure on child outcome (Ulmer-Yaniv et al., 2017). Several studies assessed links between parenting style and children's HCC, as well as concordance between maternal and child HCC, but results are inconclusive. Harsh parenting was negatively associated with 6-year-old's HCC, but only among those preterm and low birthweight children (Windhorst et al., 2017) and negative correlations were reported between HCC and positive parenting in 4-year-old children (Simmons et al., 2019). In other studies, no significant associations were found between parenting and children's HCC in refugee preschoolers (Lembcke, Buchmüller, & Leyendecker, 2020) or in a community sample of 2-year-old children (Bryson et al., 2021a). With regards to maternal and child's HCC concordance, poor parenting moderated the association between HCC in mothers and their 7-year-old daughters (Ouellette et al., 2015), whereas another study indicated that greater maternal sensitivity linked with stronger mother–child HCC concordance among 4–5-year-olds (Schloß et al., 2019). Maternal psychological symptoms have similarly been shown to correlate with children's HCC in some studies (Halevi et al., 2017) but not others, and, overall, positive, negative, and no associations were reported between maternal symptoms and children's HCC (Bryson et al., 2021a, 2021b). Since most studies targeted infants and toddlers, testing HCC in relation to adolescents’ mental health, parental psychopathology, and parenting behavior in the context of trauma exposure is required (Bryson, Goldfeld, Price, & Mensah, 2019; Flom, St. John, Meyer, & Tarullo, 2017; Galbally, van Rossum, Watson, de Kloet, & Lewis, 2019; Gray et al., 2018). Another point to consider is the minimal attention directed to the role children play in shaping their parents’ physiology and behavior, despite evidence suggesting that mother and child exert bidirectional influences on each other's condition, particularly in the context of trauma (Yirmiya, Motsan, Kanat-Maymon, & Feldman, 2021). While it has shown that maternal HCC can explain variance in children's psychopathology above and beyond children's HCC (Lembcke et al., 2020), the opposite direction, from child psychiatric condition to maternal HCC, has so far not been reported.

Although chronic stress and trauma have been linked with alterations in HPA-axis functioning across the lifespan, some developmental stages are more susceptible to the effects of trauma exposure due to the rapid maturation of stress-management support systems (Danese & McEwen, 2012). During the transition to adolescence, there is a marked increase in rates of psychopathology (Kessler et al., 2005; Merikangas et al., 2010), and the transition is especially risky for children who are growing up in stressful environments (LeMoult et al., 2020; Rudolph & Flynn, 2007), partially due to the heightened stress reactivity that characterizes the onset of puberty (Busso, McLaughlin, & Sheridan, 2017; Dahl & Gunnar, 2009; Doom & Gunnar, 2013). Yet, the transition to adolescence is also a period of increased opportunities for positive growth (Crone & Dahl, 2012), and the biobehavioral reorganization that takes place during that time may enable some youth to emerge from the transition with greater resilience. Despite the importance of the adolescent transition to well-being and adaptations throughout life (Johnson, Dupuis, Piche, Clayborne, & Colman, 2018; Patton et al., 2014), little longitudinal research described factors that may mediate the continuity of risk *v.* the emergence of resilience across the transition to adolescence or tested specific biological and relational factors that may augment or buffer the effects of stressful early environment on children's mental health.

In the current study, we utilized a cohort of children exposed to chronic war-related trauma since birth who were followed in our lab from early childhood to late adolescence. Prior reports from this cohort have shown that across the three assessment points in the first decade of life, war-exposed children presented more psychiatric disorders compared to non-exposed controls. Furthermore, it was found that maternal and child factors measured in early and late childhood, such as maternal post-traumatic stress symptoms (PTSS), sensitive caregiving, and child cortisol, shaped the trajectories of risk and resilience across the 10-year span (Halevi et al., 2017; Halevi, Djalovski, Vengrober, & Feldman, 2016).

Consistent with our conceptual model on resilience (Feldman, 2020, 2021), which considers the coordination of biological and behavioral processes in mother and child as an important mechanism by which mother externally regulates her child's psychophysiological systems and tunes him to social life, the current study aimed to describe key factors associated with resilience, particularly physiological systems implicated in the management of stress and sensitive parenting. Here, we utilized data from our cohort of war-exposed children and their mothers collected during the three assessment points in the second decade of life; late childhood, early adolescence, and late adolescence, in order to frame the adolescent transition. We aimed to pinpoint maternal and child biobehavioral factors that may mediate and moderate the chronicity of psychopathology *v.* resilience across the transition from late childhood, before the onset of the transition, to late adolescence, following the transition. Consistent with findings from previous assessments of this cohort during the first decade (Halevi et al., 2016), we hypothesized that trauma-exposed children will display more psychopathologies in late adolescence and that exposed mothers and children will have higher HCC levels compared to controls. Furthermore, we hypothesized that trauma exposure, sensitive caregiving and chronic stress physiology as indexed by maternal and child HCC will influence the continuity of psychopathology across the transition to adolescence. Specifically, we hypothesized three main pathways affecting the continuity of psychiatric disorders: (1) Exposed mothers will be less sensitive and this will moderate the continuity of psychiatric disorders so that children with psychiatric diagnosis in late childhood who are reared by less sensitive mothers will be at a greater risk to exhibit a psychiatric diagnosis in late adolescence. (2) Maternal PTSS will mediate the continuity of the child's psychiatric condition from late childhood to late adolescence. (3) Trauma exposure will moderate the association between children's diagnosis in late childhood and mothers' HCC, such that exposed mothers whose children are diagnosed with an internalizing disorder would show elevated HCC levels. Furthermore, maternal HCC will be positively associated with children's HCC, which would link with greater prevalence of maternal PTSS in early adolescence, and this in turn, will be associated with child psychopathology at late adolescence.

## Methods

### Participants

Participants were recruited during 2004–2005 and included 232 children and their mother (*M*_age_ = 2.76 years ± 0.91, 47.6% males and 47.1% firstborns). Of these, 148 dyads were living in the same front-line neighborhoods in Sderot, Israel, and comprised the trauma-exposed group. A comparison group of 84 dyads was recruited from comparable towns, matched for sociodemographic variables. The trauma-exposed group from Sderot comprised families who live near the Gaza strip border and have been exposed to repeated and unpredictable missile and rocket attacks, as well as several military operations over the past 20 years. During these attacks, which last from several days to several months, siren warning of incoming missiles are heard, sometimes dozens of times per day, allowing citizens 7–15 s to reach shelter before explosion. Sporadic rocket and missile attacks are also common during relatively calm periods. Overall, many citizens from this area have suffered from physical injuries, as well as significant property and infrastructure damage, leading to severe psychological distress among this population.

Results from early and middle childhood (*M*_age_ = 7.68 years ± 0.7) are described elsewhere (Feldman & Vengrober, 2011; Feldman, Vengrober, & Ebstein, 2014; Feldman, Vengrober, Eidelman-Rothman, & Zagoory-Sharon, 2013). Here, we use data from late childhood, early adolescence, and late adolescence. At late childhood, 177 families of the initial sample were visited at their homes (*M*_age_ = 9.3 years ± 1.41; 101 war-exposed, 76 controls) and underwent psychiatric evaluation and observations. At early adolescence, 111 mother–child dyads visited the laboratory (*M*_age_ = 11.66 years ± 1.23; 58 war-exposed, 53 controls). At late adolescence data from 138 families (64 males) were home-visited (*M*_age_ = 15.65 years ± 1.31; 70 war-exposed, 68 controls). Attrition was mainly related to inability to locate families or families moving out of Sderot. No differences in child age, child sex, maternal education, maternal age, and family socioeconomic status (SES) were found between continuing or non-continuing families. The study was approved by the University's Institutional Review Board, and all parents signed informed consent.

### Procedures

#### Late childhood

**Fig. 1.**
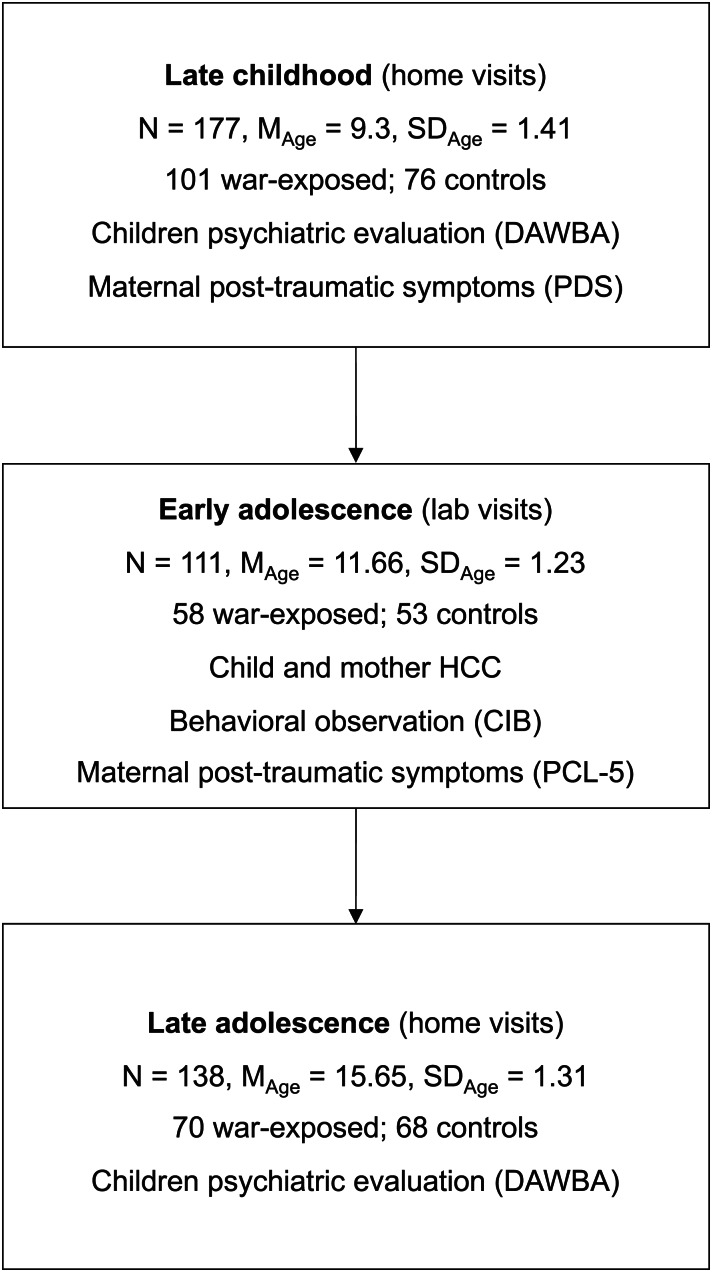
Time-line and study variables from late childhood to late adolescence. *Note*: Child diagnoses at late childhood and late adolescence were evaluated using The Developmental and Well-Being Assessment (DAWBA); maternal sensitivity was evaluated using the Coding Interactive Behavior (CIB). HCC, hair cortisol concentration; PDS, Post-Traumatic Diagnostic Scale; PCL, Post-Traumatic Stress Checklist.

Mothers and children were visited at home and engaged in interaction paradigm in which they were asked to plan the ‘best day ever’ to spend together for 7 min (Halevi et al., 2017; Ulmer-Yaniv et al., 2017). Mothers and fathers completed psychological questionnaires regarding themselves and their child and mothers completed the Developmental and Well-Being Assessment (DAWBA) through interview by a trained psychologist. (Study time-line and participants are presented in Fig. 1.)

#### Early adolescence

A lab visit included interviews, hormonal assessment, and relational and individual paradigms. Dyads were asked to play ‘Etch a Sketch’ game for 7 min. This is a game in which mother and child each control one of two knobs that enable the drawing of either vertical or horizontal lines. Dyads need to coordinate their activity to create a drawing. Mothers and children completed psychological questionnaires.

#### Late adolescence

Home visits were held in which trained clinicians re-evaluated the subjects' psychiatric condition using the DAWBA.

### Measures

#### Child psychiatric diagnosis

The DAWBA was used to diagnose children's Axis-I internalizing disorders at late childhood and late adolescence. The DAWBA is a structured interview generating ICD-10 and DSM-IV psychiatric diagnoses in 5–17-year-old children (Goodman, Ford, Richards, Gatward, & Meltzer, 2000). The DAWBA, administered to mothers, is well-validated, including a large epidemiological study in Israel (Mansbach-Kleinfeld, Apter, Farbstein, Levine, & Poznizovsky, 2010). The DAWBA was administered by clinical psychologists and supervised by the same child psychiatrist, blind to all other information, at both stages. Cases were conferred every few weeks with reliability exceeding 85%.

#### Hair cortisol concentrations

At early adolescence, hair strands were cut as close as possible to the scalp from a posterior vertex position, and the first 3 cm of hair cut proximal to the scalp were analyzed. Hair samples were stored in an envelope in the dark at room temperature until assayed. We extracted steroids from hair using our published protocol for hair-testing (Schonblum et al., 2018). Briefly, hair was weighed and placed in a glass vial. Methanol was added and the vials were sonicated for 30 min and then incubated overnight at 50 °C with gentle shaking. The methanol was collected and evaporated under a stream of nitrogen. Samples were reconstituted in 10% methanol and 90% assay diluent that was provided with the commercial enzyme-linked immunosorbent assays (ELISA) according to manufacturer's recommendations. HCC was quantified in hair extracts using commercial ELISA according to the manufacturer's recommendations (Salimetrics; item no. 1-3002-5; Ann Arbor, MI, USA). Serial dilutions of separate pools for mother and children showed parallelism with the provided kit standards (univariate analysis of variance in SPSS; *p* = 0.02).

Linearity was demonstrated for all the weight range examined, between 10 and 140 mg of hair extract, we therefore used 10–80 mg of hair for cortisol extraction. According to the manufacturer, antibody cross-reactivity was reported as 19.2% with dexamethasone and less than 0.568% with all other steroids. Intra-assay variability was determined using six duplicates of the pool on the same ELISA plate (CV; 5.26%). Inter-assay precision was determined by running duplicates of the pool on four different days (CV; 4.75%). Recovery was estimated by the addition of a known amount of cortisol standard to the hair extract (105.5%).

#### Maternal sensitivity

Early adolescence interactions were coded using the Coding Interactive Behavior Manual (CIB) (Feldman, 1998). The CIB is a well-validated tool to evaluate social behavior across ages and has shown good psychometric properties across different ages and cultures. Interactions are coded on multiple global scales from 1 (low) to 5 (high) based on frequency, intensity, and duration of each behavior or social orientation. Maternal sensitivity included the codes of maternal acknowledgment, appropriate range of affect, containment, supportive presence, and empathy. Two trained coders, blind to other information, coded the interactions and reliability on 20% of the interactions exceeded 90% on all codes (*k* > 0.82, range 0.78–96). Variables were averaged to create the maternal sensitivity score (Cronbach's *α* = 0.91).

#### Maternal PTSS

In late childhood, mothers completed the Post-traumatic Diagnostic Scale (PDS), a 17-item self-report questionnaire for assessing PTSS based on DSM-IV criteria (Foa, Cashman, Jaycox, & Perry, 1997). Each item is scored on a scale of 0–3 and all symptoms are summed to create a total severity score (range 0–51), with higher scores indicating greater PTSS. At early adolescence, mothers completed the fifth version of the PTSD Checklist (PCL-5) (Weathers et al., 2013), a 20-item self-report designed to assess the DSM-5 symptoms of PTSD. Respondent rated on a five-point Likert scale distress associated with each symptom to a total symptom severity score (range 0–80) obtained by summing the 20 items.

### Statistical analysis

We first examined differences between exposed and non-exposed groups in demographic condition and study variables using *t* tests, χ^2^ and McNemar's tests. Hair cortisol was positively skewed and Box–Cox transformed (Osborne, 2010). For a comprehensive model on the direct, mediated, and moderated paths from child symptomatology at late childhood to late adolescence, as mediated and moderated by war-exposure, maternal and child HCC, maternal sensitivity and PTSS, we conducted structural equation model (SEM) with Amos 21.0 (Arbuckle, 2012; Byrne, 2016). We ran two identical models; one for internalizing and one for externalizing diagnoses. All variables in the models were standardized using *z*-transformation. To assess model fit the following indices were used: χ^2^, comparative fit index (CFI), Bollen's incremental fit index (IFI), and the root mean square error of approximation (RMSEA). CFI and IFI ≥0.90 and RMSEA ≤0.08 valeus are considered to indicate a good fit (Hu & Bentler, 1999). Ideally, the χ^2^ statistic is expected to be non-significant in the case of adequate fit, however this index is no longer used to evaluate fit because of its hypersensitivity to sample size (Hu & Bentler, 1999). Full information maximum-likelihood method accounted for missing data was estimated in all analyses. Children's age and gender were inserted as covariates. An additional model included maternal PTSS in late childhood as a covariate, to test the inference of the longitudinal ordering and effects, as suggested by Cole and Maxwell (2003). Significance of moderation effects, simple slopes and confidence intervals (CI) were computed with PROCESS for SPSS (v. 3.3) (Hayes, 2013). PROCESS employs bootstrapping calculations, which provides the most powerful method for defining confidence limits for conditional indirect effects at different levels of the moderators and accounts for violation of normality that can occur in more traditional approaches (Hayes, 2022). Bias-corrected standard errors and CIs were generated using 5000 bootstrapped resamples drawn to derive the 95% CI. Conditional mediation is present when the CI for the estimation of indirect effect does not contain zero. We used PROCESS Model 1 to test each moderation effect, and compared the effect size and s.e. from the AMOS and PROCESS analyzes to ascertain consistence across methods. To test the presence of mediation paths from child diagnosis in late childhood to diagnosis in late adolescence via mothers' and children's HCC and maternal PTSS we used the ‘Mediation’ package within the R statistical software (Tingley, Yamamoto, Hirose, Keele, & Imai, 2014). This package enables to test the indirect effects using the Monte Carlo CI for complex functions method (Tofighi & MacKinnon, 2016). Two children's hair samples were excluded as HCC data were extreme outliers (>3 standard deviations above the mean). We included in our final model behavioral/psychological assessments and hair samples from 78 mothers and 53 children.

## Results

### Sample characteristics and child psychopathology in war-exposed and control

**Fig. 2.**
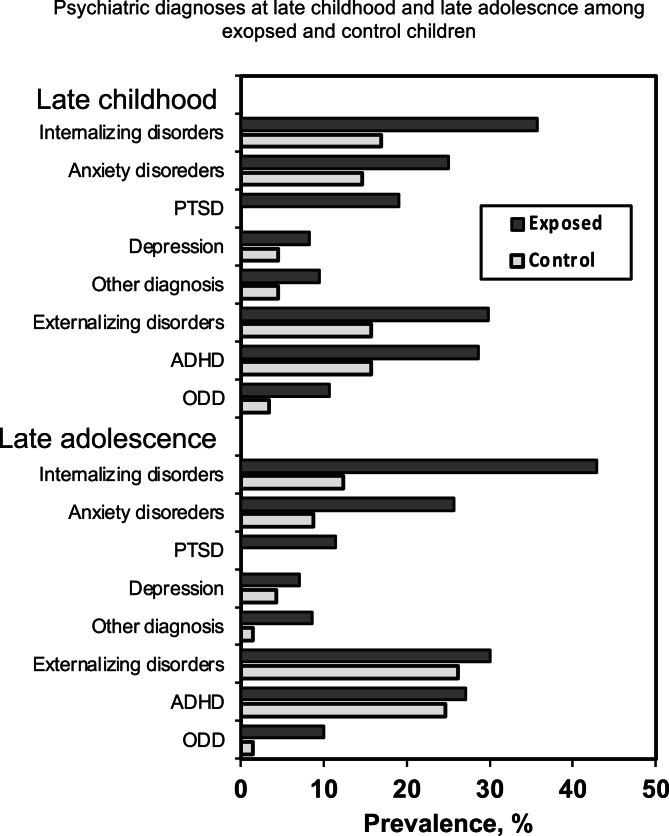
Prevalence of psychiatric disorders in late childhood and late adolescence. *Note*: Significant difference in overall rate of internalizing diagnoses between exposed and control children at late childhood [χ^2^_(1)_ = 7.99, *p* = 0.005] and late adolescence [χ^2^_(1)_ = 15.55, *p* < 0.001]; significant between-group difference in anxiety disorders at late adolescence [χ^2^_(1)_ = 6.26, *p* = 0.012]; PTSD late childhood [χ^2^_(1)_ = 18.68, *p* < 0.001]; PTSD late adolescence [χ^2^_(1)_ = 7.90, *p* = 0.005]; ADHD late childhood [χ^2^_(1)_ = 4.16, *p* = 0.041]; ODD late adolescence [χ^2^_(1)_ = 4.33, *p* = 0.037].

Detailed sample characteristics by exposure are provided in Table 1. No differences in child age, child gender, maternal age and family SES were found between the exposed and control groups (*p* > 0.05 for all variables). Results presented in Table 1 and Fig. 2 show that at late childhood and late adolescence war-exposed children had more internalizing psychopathologies, and at early adolescence exposed mothers had more PTSS and were less sensitive than non-exposed mothers. Children's main internalizing disorders in a descending prevalence at late childhood included specific phobia, PTSD, anxiety NOS, generalized anxiety disorder, separation anxiety, and depression. At late adolescence internalizing disorders in a descending prevalence were specific phobia, anxiety NOS, generalized anxiety disorder, PTSD, social phobia, separation anxiety, social anxiety, and depression. Group differences in main psychopathologies at each time-point are presented in Fig. 2. There was a 4.2% increase in internalizing pathologies from late childhood to late adolescence for the whole sample (from 30.6 to 34.8%). Among exposed children, prevalence of internalizing disorders increased from 42.9% at late childhood to 51.4% at late adolescence, describing an 8.5% increase, while among controls, internalizing disorders decreased from late childhood to late adolescence (from 19.1 to 17.6%). McNemar's test for paired nominal data yielded non-significant results (which may be related to missing data), nor were there differences in the type of specific psychopathologies at the two ages (see Fig. 2).

**Table 1.** Group differences among study variables

	Control		*N*	Exposed		*N*	
	*M*/%	s.d.		*M*/%	s.d.		
Sociodemographic variables							
Gender (female)	57.0%		54	45.3%		39	χ^2^_(1)_ = 2.42, *p* = 0.120
Child age T5	15.66	1.20	68	15.30	1.44	70	*t*_(__136)_ = −1.20, *p* = 0.232
Mother age T5	42.53	4.50	62	41.76	5.58	68	*t*_(__128)_ = 0.86, *p* = 0.393
Family SES (1–7)	4.18	1.41	68	4.14	0.99	68	*t*_(__134)_ = 0.14, *p* = 0.889
Late childhood							
Child internalizing psychopathology	19.1%		17	42.9%		36	χ^2^_(1)_ = 11.48, *p* = 0.001
Maternal post-traumatic symptoms (PDS)	1.23	3.41	88	5.69	9.55	82	*t*_(__168)_ = −4.00, *p* < 0.001
Early adolescence							
Child HCC (pg/mg)	2.35	2.75	28	2.88	3.39	25	*t*_(__51)_ = −0.63, *p* = 0.533
Mother HCC (pg/mg)	4.92	4.46	42	6.98	5.74	36	*t*_(__76)_ = −1.79, *p* = 0.077
Maternal sensitivity	3.40	1.02	49	2.93	0.93	51	*t*_(__98)_ = 2.42, *p* = 0.017
Maternal post-traumatic symptoms (PCL-5)	6.69	11.14	49	20.40	17.58	57	*t*_(__104)_ = −4.86, *p* *<* 0.001
Late adolescence							
Child internalizing psychopathology	17.6%		12	51.4%		36	χ^2^_(1)_ = 17.35, *p* < 0.001

Internalizing psychopathology was measured using the Developmental and Well-Being Assessment DAWBA, hair cortisol concentrarion (HCC) was Box–Cox transformed, maternal sensitivity was measured using the Coding Interactive Behavior Manual (CIB), and maternal post-traumatic symptoms were measured at late childhood using the Post-Traumatic Diagnostic Scale (PDS) and at early adolescence using the PTSD Checklist (PCL-5).

### Cortisol levels

Hair cortisol levels were between 0.01 and 11.90 pg/mg for children (*M* = 2.60 ± 3.05) and 0.01 and 24.01 pg/mg for mothers (*M* = 5.88 ± 5.16) for the entire sample. There was a trend toward higher HCC in exposed mothers (*p* = 0.07), but not in the children (see Table 1). No correlations emerged between HCC and children's age [*r*_(51)_ = 0.02, *p* = 0.88] and no gender differences [t_(51)_ = −0.45, *p* = 0.65]. Mothers' and children's HCC was inter-related [*r*_(40)_ = 0.52, *p* < 0.001].

### HCC, maternal sensitivity, and PTSS impact internalizing disorders in adolescence

**Fig. 3.**
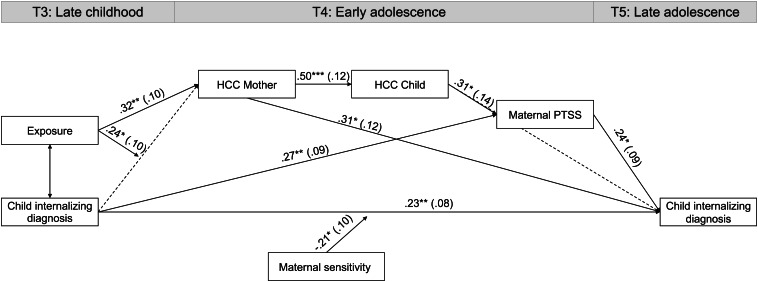
Path model leading from child internalizing disorder at late childhood to child internalizing disorder at late adolescence via three mediating and moderating paths of maternal sensitivity, maternal post-traumatic stress symptoms, and child and mother HCC. *Note*: Coefficients represent standardized regression weights and standard errors. *p < 0.05, **p < 0.01, ***p < 0.001. ^ǂ^Controlling for child age and child gender. Child diagnoses at late childhood and late adolescence were evaluated using The Developmental and Well-Being Assessment (DAWBA); maternal sensitivity was evaluated using the Coding Interactive Behavior (CIB); maternal PTSS were evaluated in early adolescence using the Post-Traumatic Stress Checklist (PCL-5). HCC, hair cortisol concentration; PTSS, post-traumatic stress symptoms.

SEM tested our model on the mediating and moderating role of war-exposure, HCC, maternal sensitivity, and PTSS in the association between internalizing disorders across the adolescent transition, from late childhood to late adolescence. The overall model provided good fit to the data [χ^2^_(9)_ = 18.12, *p* = 0.034, RMSEA = 0.06, CFI = 0.92, IFI = 0.91]. The final path model is presented in Fig. 3: child psychiatric diagnosis at late childhood linked with child psychiatric diagnosis at late adolescence via three main paths:

#### Maternal sensitivity moderates the continuity of child diagnosis

Links between child diagnosis at late childhood and late adolescence were moderated by maternal sensitivity (coefficient = −8.23, s.e. = 0.40, 95% CI −1.62 to −0.02). Child diagnosis at late childhood predicted diagnosis at late adolescence, but only for children with less sensitive mother. To further probe this indirect effect, we evaluated this path at high (1 s.d. above mean) and low (1 s.d. below mean) levels of maternal sensitivity (Hayes, 2018). A significant simple slope emerged for low maternal sensitivity (*B* = −1.86, s.e. = 0.56, 95% CI 0.75–2.96), but when sensitivity was high, the simple slope was non-significant (*B* = 0.25, s.e. = 0.41, 95% CI −0.56–1.07). This indicates that for children with an internalizing diagnosis in late childhood, the likelihood to be diagnosed also at late adolescence was higher for children who had less sensitive mothers.

#### Maternal PTSS mediates continuity of child psychopathology

The second pathway involved mediation; child diagnosis at late childhood linked with higher maternal PTSS, which in turn predicted greater prevalence of internalizing disorders at late adolescence (estimate: 0.08, 95% CI 0.008–0.14).

#### Exposure, HCC, and maternal PTSS

**Fig. 4.**
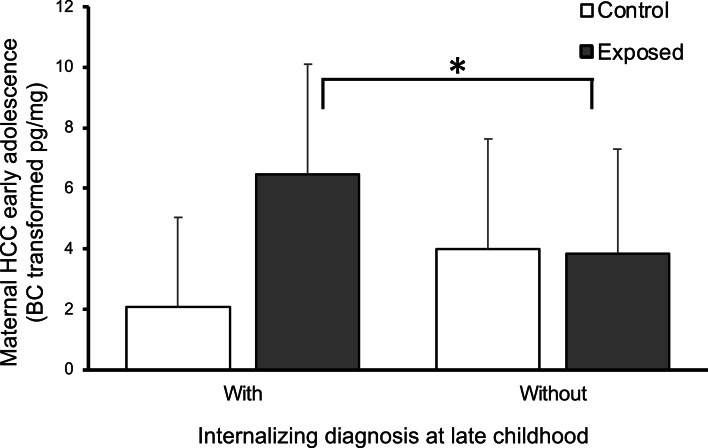
The effect of internalizing diagnosis at late childhood and exposure in predicting mother's HCC levels at early adolescence: Internalizing disorder at late childhood predicted mothers' higher HCC at early adolescence for the exposed group, but not for the control group. *Note*: Child internalizing diagnosis was evaluated using The Developmental and Well-Being Assessment (DAWBA). HCC, hair cortisol concentration.

The third pathway involved a moderation-mediation path in which war exposure moderated the link between child diagnosis and maternal HCC. This pathway began with an interaction effect (moderation), which revealed that HCC was highest among war-exposed mothers whose child showed psychiatric diagnosis at late childhood. This moderation effect was significant (coefficient = 0.28, s.e. = 0.11, 95% CI 0.05–0.51), and can be seen in Fig. 4. Evaluation of this moderation effect revealed significant positive simple slope for exposed (*B* = 0.32, s.e. = 0.15, 95% CI 0.01–0.62), but non-significant simple slope for controls (*B* = −0.23, s.e. = 0.17, 95% CI −0.58–0.10), indicating that only among exposed families a significant association between child's psychiatric diagnosis at late childhood and maternal HCC was found. The mediation pathway included maternal HCC, which was correlated with child HCC, later linked with maternal PTSS, which in turn predicted greater risk for psychiatric diagnosis in late adolescence. Test of mediation showed that this indirect path from child diagnosis at late childhood to late adolescence via mother and child HCC, and maternal PTSS was significant (estimate: 0.01, 95% CI 0.001–0.03). In this pathway, child diagnosis in late childhood (*x*) predicted child diagnosis at late adolescence (*y*) via exposure (moderator), maternal and child HCC (mediators), and maternal PTSS (mediator). A similar indirect pathway began with the same moderation effect, in which war exposure moderated the link between child diagnosis at late childhood and maternal HCC at early adolescence. Maternal HCC, in turn, positively predicted child's diagnosis at late adolescence (estimate: 0.06, 95% CI 0.007–0.18). Adding maternal PTSS in late childhood as a covariate did not significantly change the estimates and s.e.; however, this model provided a less adequate fit to the data (see online Supplementary Fig. S1).

### Testing paths leading from HCC, maternal sensitivity, and mothers’ PTSS I to adolescents’ externalizing disorders

To ascertain that the pathway is specific to internalizing pathology, we tried to fit externalizing disorders instead of internalizing disorders to the suggested model and found that most pathways were not significant, possibly due to the strong direct association between externalizing disorders in late childhood and externalizing disorders in late adolescence. Furthermore, the alternative externalizing model did not provide an adequate fit to the data (see online Supplementary Fig. S2), leading to the conclusion that the mediators and moderators examined here apply specifically to the continuity of internalizing disorders.

## Discussion

The adolescent transition marks a period of great plasticity associated with increased risk as well as enhanced opportunities for resilience. Our study focuses on the role of maternal and child's physiological stress response, sensitive caregiving, and the mothers’ post-traumatic symptomatology as moderators and mediators of the continuity of risk *v.* resilience across the transition to adolescence in children exposed to a specific and chronic trauma (Halevi et al., 2017; Ulmer-Yaniv et al., 2017; Yirmiya, Djalovski, Motsan, Zagoory-Sharon, & Feldman, 2018). To our knowledge, this is the first study that examines the long-term effects of trauma exposure on adolescents’ mental health which utilizes measures of maternal and child HCC. Overall, our sample provides a unique ‘natural experiment’ to study the long-term effects of trauma, as all children in the study were exposed to the exact same stressors, a rare condition in ELS and trauma-exposure research, and this opportunity may help shed further light on the direct and indirect pathways to psychopathology and resilience across a key developmental transition.

Our results indicate that more than 60% of trauma-exposed children exhibited at least one full-blown DSM internalizing disorder in late childhood and/or late adolescence, as compared to only 25% of controls, highlighting the immense burden of growing up in a harsh, unpredictable, and traumatic environment. Moreover, while only 4.2% of the control adolescents displayed unremitted psychopathology across the adolescent transition, more than half of the war-exposed children who suffered from an internalizing diagnosis in late childhood remained symptomatic. These findings on the chronicity of war-related psychopathologies accord with research in younger children and adults (Feldman et al., 2014; Halevi et al., 2016; Hobfoll et al., 2009), but few, if any, studies tested such chronicity across the transition to adolescence. It appears that prolonged early stress leads to early-onset psychopathology that is characterized by a persistent course, with few opportunities for recovery. Still, even in such a harsh context we found that some children exhibited resilience to chronic stress exposure and the results pinpoint the interplay between maternal and child biobehavioral mediators and moderators of resilience.

Maternal sensitivity moderated the continuity of internalizing disorders from late childhood to late adolescence. Overall, exposed mothers were less sensitive compared to mothers of control children, consistent with prior research on the reduction in sensitive parenting in the context of early adversity (Creech, Hadley, & Borsari, 2014; Kelley et al., 2010; Lewig, Arney, & Salveron, 2010; Ulmer-Yaniv et al., 2017; Yirmiya et al., 2018). Our results suggest that only mothers who were sensitive throughout the transition showed a stress-buffering function and decreased the risk of persistent psychiatric diagnosis across the adolescent transition. Our findings also accord with studies on the concurrent and longitudinal relations between maternal insensitivity and the development of children's internalizing problems across childhood and adolescence (Feldman, 2010; Feldman & Eidelman, 2004; Kok et al., 2013; Mäntymaa et al., 2009; van der Voort et al., 2014).

Inhibited and withdrawn parental behaviors in reaction to adolescent-related stressors were found to pose a risk for depression (Buck & Dix, 2012). Sensitive mothering may protect specifically against such age-specific stressors since research shows that behavioral inhibition in middle childhood mediated the relations between maternal sensitivity and internalizing problems in adolescence (van der Voort et al., 2014). Adolescents' emotional insecurity, expressed in the parent–child relationship, may be another mediator between maternal warmth and internalizing problems in adolescence, since mental representations of the attachment relationship is important for expanding the adolescent's social world (Alegre, Benson, & Pérez-Escoda, 2014). Despite the adolescent's growing autonomy, studies have shown that adolescents still rely on closeness with their parents (Lieberman, Doyle, & Markiewicz, 1999; Yirmiya et al., 2021). Consistently, maternal sensitivity has been found to predict internalizing problems in prepubertal and post-pubertal children (Haltigan, Roisman, Cauffman, & Booth-LaForce, 2017); maternal attunement moderated the effect of mother's parenting stress on adolescents' internalizing problems (Arbel et al., 2020); and maternal responsiveness and autonomy support have been shown to predict adolescents' attachment style, which in turn linked with internalizing symptoms (Brenning, Soenens, Braet, & Bal, 2012).

Maternal post-traumatic symptomatology mediated the link between children's internalizing diagnosis across the transition to adolescence, and this path was significant both directly and indirectly via mother and child's HCC. The co-occurrence of mothers’ and children's psychiatric symptoms following trauma has been repeatedly reported (Leen-Feldner et al., 2013; Smith, Perrin, Yule, & Rabe-Hesketh, 2001; Thabet, Tawahina, El Sarraj, & Vostanis, 2008; Yirmiya et al., 2021); however, most studies did not test longitudinal associations, measured bidirectional effects, or focused on adolescence. The few existing longitudinal studies showed that maternal PTSD chronicity predicted higher PTSD and anxiety among adolescents exposed to earthquake 10 years earlier (Chen et al., 2020). Maternal PTSD was also found to mediate the association between hurricane-related trauma exposure and adolescents’ internalizing symptoms (Spell et al., 2008). These studies, combined with the current results, highlight the mother's psychopathology as a risk factor in mental health outcome of disaster-exposed even when controlling for trauma exposure.

The transmission between mothers' and children's psychiatric symptoms following trauma could be attributed to genetic factors (Sartor et al., 2012). Such genetic vulnerability may increase the risk for trauma-related psychopathologies directly in both mother and child, or indirectly, via the dyadic relationship and behaviors secondary to parental psychopathology. The association between parent's and offspring's trauma-related symptoms may also stem from shared environmental factors; for instance, correlations between parent and child's depressive symptoms were found among genetically-related and unrelated dyads, which were not explained by shared adversity factors, such as negative life events or family income (Lewis, Rice, Harold, Collishaw, & Thapar, 2011). Furthermore, in cohorts such as ours, where the whole family is exposed to the same stressor, reciprocal influences between children and parents can be expected. This phenomenon, termed the ‘compound effect’, describes situations in which families face trauma together and the symptoms of each family member influence and intensify those of other members (Scheeringa & Zeanah, 2001). Maternal PTSS may also relate to pathological alterations in the mother's stress response, which may link with genetic or epigenetic transmission of vulnerability to her children. Traumatic experiences and other stressors may lead to changes in stress biomarkers, such as HCC, and these may depend on the shared mother–child genotype (Koenig et al., 2018).

The mother's stress-management systems in general, and HPA-axis functioning in particular, have been extensively studied as a stress-buffering mechanism for the offspring's hormonal and behavioral regulation (Hostinar, Sullivan, & Gunnar, 2014). Here, the highest levels of maternal HCC in early adolescence were among war-exposed mothers whose children suffered from internalizing disorders in late childhood, pointing to the cumulative effects of chronic stressors on maternal HPA-axis regulation. Moreover, mothers' HCC predicted both directly and indirectly, via the child's HCC and post-traumatic symptoms, the child's psychiatric diagnosis in late adolescence. Evidence from animal studies indicates that maternal HPA-axis shapes the offspring's stress response even before birth and mediates the effects of multiple contextual and psychological stressors on the maturation of stress reactivity (Barbazanges, Piazza, Le Moal, & Maccari, 1996; Dinces, Romeo, McEwen, & Tang, 2014; Seckl & Meaney, 2004). In humans, maternal HPA regulation during pregnancy shapes the infant's stress reactivity and physiological well-being (Davis, Glynn, Waffarn, & Sandman, 2011; Graham et al., 2019; Khalsa et al., 2018), and maternal HPA-axis regulation during gestation predicts later child internalizing problems (Graham et al., 2019). Although much research has been conducted on the role of maternal–infant bonding in shaping infant stress reactivity, much less attention has been directed to adolescence in general and the adolescent transition in particular (Hostinar et al., 2014). Furthermore, at both late childhood (Halevi et al., 2017) and early adolescence, a strong dyadic HCC linkage was found, which reflects the coupling of maternal and child physiological responses. Since the mother signals environmental threats to her offspring, such linkage may be an important survival-related mechanism that contributes to infant adaptation and resilience.

In contrast to our hypothesis, we did not find significant differences between exposed and control participants' HCC levels. While some studies reported a decrease in HCC following trauma-exposure (Buchmüller et al., 2020; Steudte et al., 2013), most studies reported elevated HCC levels among trauma-exposed adults (Mewes, Reich, Skoluda, Seele, & Nater, 2017; Schumacher et al., 2022; Stalder et al., 2016), and in our cohort there was a trend toward higher HCC among exposed mothers. Children's HCC levels showed no differences between the exposed and control groups, which is consistent with another study on a similar cohort of war-exposed youth (Shaheen et al., 2020). Such lack of difference is surprising, and suggests that the effects of trauma exposure on HCC are indirect and other factors mediate this association. One possible mediator is the type of psychiatric diagnosis; war-exposed participants varied in their psychiatric diagnoses, and previous studies demonstrated that some of these psychiatric conditions can influence HCC in opposite directions (Koumantarou Malisiova et al., 2021; Staufenbiel et al., 2013).

In sum, the current study is first to examine maternal and adolescent's HCC, maternal PTSS, and parenting behavior as mediators and moderators of the continuity of internalizing psychopathology across the transition to adolescence. Our cohort is unique and was followed for a lengthy period. Chronic exposure to war, terror, and trauma is known to affect children's propensity to psychopathology; yet, despite much research on the effects of and the role of HPA-axis regulation, few studies focused on the adolescent transition, which shapes mental health and adaptation throughout life (Johnson et al., 2018). Our study may therefore have a unique contribution to further understanding mechanisms transmission and determinants of resilience at the transition to adolescence.

Several study limitations should be acknowledged. These include the exclusion of fathers' stress physiology and behavior and the stress-buffering effects of other close relationships, such as siblings, peers, or grandparents. Another limitation is the lack of the participants’ subjective experiences of the trauma and the degree of stress it triggered, measures that could have provided a clearer picture on the associations between stress, HCC, behavior, and symptomology. Attrition and inability to collect HCC for all study participants is another limitation, which led to a relatively small sample size, especially in light of the complex statistical model carried out. In early adolescence, families were invited to our laboratory instead of the home visits we conducted at all other time-points and it was difficult for some families to participate at this measurement time-point. Our study may contribute to shed light on some of the roots of resilience, particularly to understanding the important role sensitive caregiving plays during adolescence and the involvement of chronic stress regulation in resilience (Feldman, 2020). Charting these pathways is essential for the construction of more effective interventions that take into account the important role of maternal physiology and behavior during the transition to adolescence.

## Supplementary material

For supplementary material accompanying this paper visit https://doi.org/10.1017/S0033291722001350. click here to view supplementary material
