# Supplementary material for: Continuity of psychopathology v. resilience across the transition to adolescence: role of hair cortisol and sensitive caregiving
Source: Psychol Med. 2022 May 30;53(10):4487–98. doi: 10.1017/S0033291722001350 (PMC10388331; doi:10.1017/S0033291722001350)
Supplement: Supplementary file 1 [file S0033291722001350sup001.docx]

Supplementary Figure 1: Path model leading from child internalizing disorder at late childhood to child internalizing disorder at late adolescence via three mediating and moderating paths of maternal sensitivity, maternal post-traumatic stress symptoms and child and mother HCC

Note: Coefficients represent standardized regression weights and standard errors. ^*^ p < .05, ^**^ p <.01, ^***^ p < .001. ǂControlling for child age, child gender and maternal post-traumatic stress symptoms at early childhood. The overall model provided an acceptable fit to the data: χ^2^_(11)_ = 26.63, p = .005, CFI = .91, NFI = .90, RMSEA = .078. Child diagnoses at late childhood and late adolescence were evaluated using The Developmental and Well-Being Assessment (DAWBA); maternal sensitivity was evaluated using the Coding Interactive Behavior (CIB); maternal PTSS were evaluated at late childhood using the Post-Traumatic Diagnostic Scale (PDS) and in early adolescence using the Post-Traumatic Stress Checklist (PCL-5). Abbreviations: HCC, Hair Cortisol concentration; PTSS, Post-Traumatic Stress Symptoms.

Supplementary Figure 2: Path model leading from child externalizing disorder at late childhood to child externalizing disorder at late adolescence via three mediating and moderating paths of maternal sensitivity, maternal post-traumatic stress symptoms and child and mother HCC

Note: Coefficients represent standardized regression weights and standard errors. ^*^ *p* < .05, ^**^ *p* <.01, ^***^ *p* < .001. ǂControlling for child age and gender. The overall model did not provide an adequate fit to the data: *χ^2^_(9)_* = 35.58, *p* < .001, CFI = .81, NFI = .86, RMSEA = .11. Child diagnoses at late childhood and late adolescence were evaluated using The Developmental and Well-Being Assessment (DAWBA); maternal sensitivity was evaluated using the Coding Interactive Behavior (CIB); maternal PTSS were evaluated in early adolescence using the Post-Traumatic Stress Checklist (PCL-5). Abbreviations: HCC, Hair Cortisol concentration; PTSS, Post-Traumatic Stress Symptoms.
